# Reducing defects in the datasets of clinical research studies: conformance with data quality metrics

**DOI:** 10.1186/s12874-019-0735-7

**Published:** 2019-05-10

**Authors:** Naila A. Shaheen, Bipin Manezhi, Abin Thomas, Mohammed AlKelya

**Affiliations:** 1 Department of Biostatistics and Bioinformatics, King Abdullah International Medical Research Center, P.O. Box 22490, Mail Code 1515, Riyadh, 11426 Kingdom of Saudi Arabia; 2 Research Quality Management Section, King Abdullah International Medical Research Center, Riyadh, Kingdom of Saudi Arabia; 3 King Saud bin Abdulaziz University for Health Sciences, Riyadh, Kingdom of Saudi Arabia; 4 Ministry of National Guard-Health Affairs, Riyadh, Kingdom of Saudi Arabia; 5 Center for Health Research Studies, Saudi Health Council, Riyadh, Kingdom of Saudi Arabia; 6 Public Health Division, Central Australian Aboriginal Congress, Alice Springs, Australia

**Keywords:** Defective dataset, Data entry errors, Clinical research data quality, Data quality metrics, Poor-quality dataset, Data quality management

## Abstract

**Background:**

A dataset is indispensable to answer the research questions of clinical research studies. Inaccurate data lead to ambiguous results, and the removal of errors results in increased cost. The aim of this Quality Improvement Project (QIP) was to improve the Data Quality (DQ) by enhancing conformance and minimizing data entry errors.

**Methods:**

This is a QIP which was conducted in the Department of Biostatistics using historical datasets submitted for statistical data analysis from the department’s knowledge base system. Forty-five datasets received for statistical data analysis, were included at baseline. A 12-item checklist based on six DQ domains (i) completeness (ii) uniqueness (iii) timeliness (iv) accuracy (v) validity and (vi) consistency was developed to assess the DQ. The checklist was comprised of 12 items; missing values, un-coded values, miscoded values, embedded values, implausible values, unformatted values, missing codebook, inconsistencies with the codebook, inaccurate format, unanalyzable data structure, missing outcome variables, and missing analytic variables. The outcome was the number of defects per dataset. Quality improvement DMAIC (Define, Measure, Analyze, Improve, Control) framework and sigma improvement tools were used. Pre-Post design was implemented using mode of interventions. Pre-Post change in defects (zero, one, two or more defects) was compared by using chi-square test.

**Results:**

At baseline, out of forty-five datasets; six (13.3%) datasets had zero defects, eight (17.8%) had one defect, and 31(69%) had ≥2 defects. The association between the nature of data capture (single vs. multiple data points) and defective data was statistically significant (*p* = 0.008). Twenty-one datasets were received during post-intervention for statistical data analysis. Seventeen (81%) had zero defects, two (9.5%) had one defect, and two (9.5%) had two or more defects. The proportion of datasets with zero defects had increased from 13.3 to 81%, whereas the proportion of datasets with two or more defects had decreased from 69 to 9.5% (*p* = < 0.001).

**Conclusion:**

Clinical research study teams often have limited knowledge of data structuring. Given the need for good quality data, we recommend training programs, consultation with data experts prior to data structuring and use of electronic data capturing methods.

## Background

Data are a fundamental resource for any health-care organization. Data are defined as “a record of signs and observations collected from various sources” [1]. A dataset is indispensable to answer the research questions of a clinical research study. According to the Joint Commission International’s *Accreditation Standards for Hospitals* (2017), study sponsors are required to comply with quality and safety criteria to ensure that the data generated are valid and that the resulting report is statistically accurate [2]*.* Maintaining data collection and entry standards is an elementary requirement of clinical research studies. Data collection standards are largely based on data elements commonly understood across clinical research. The impaired interoperability of unstandardized data hinders the exchange of clinical information between clinical researchers [3]. Data Quality (DQ) standards are essential for sharing and reusability. The clinical data quality standards must include data fields (variables) and data values (assigned codes) [4]. The clinical research data quality standards need to focus on all types of research (i.e. observational, epidemiological, interventional, and basic science research) [4].

DQ assessment is fundamental to obtain high quality data [5]. DQ, defined as its “fitness for use” [6], is a neglected consideration in many industries [7]. However, high DQ is a key ingredient for an organization’s success [8–10] and warrants prioritization [7]. Industry experts have identified gaps in DQ management. A summary of surveys conducted by Marsh et al. (2005) sheds light on the causes of poor DQ. Poor-quality or suboptimal datasets not only lead to ambiguous results, but also to repetition of the work and delayed publication. The consequences of poor DQ are customer dissatisfaction, increased project costs, lower employee performance, lower job satisfaction, and inefficacious decision-making [11, 12]. Poor DQ also causes a lack of trust in the generated data. A survey by SAS Institute (2003) reported that 67% of managers believe that poor DQ impacts customer satisfaction [13]. Studies also indicate that poor DQ contributes to 41% of project failures [14]. According to Redman [15], the total cost of poor DQ ranges from 8 to 12% of company revenues. The estimated annual cost of poor DQ in America alone is US $600 billion per year [16]. Prior to Statistical Data Analysis (SDA); datasets require cleaning and preparation by the removal of data entry errors and inconsistencies. However, operational costs increase as a result of time expenditure on error detection and data cleaning [12, 17]. The six primary dimensions of DQ reported by the Data Management Association are: (i) completeness (ii) uniqueness (iii) timeliness (iv) accuracy (v) validity; and (vi) consistency [18]. There are two aspects of DQ: quality of design and quality of conformance [16].

We report on the results of this Quality Improvement Project (QIP) that was designed to improve DQ by enhancing conformance and minimizing data entry errors. The paper is organized according to the DQ metrics for clinical research studies datasets. We summarize the results at baseline, the results after intervention to improve DQ, and the change in DQ between baseline and after intervention.

## Methods

This was a QIP designed to improve the quality of clinical research conducted by Principal Investigators (PIs) in a tertiary care hospital. The main objective of this QIP was to improve the quality of datasets generated in the clinical research by reducing the datasets errors using pre-post intervention design.

The current QIP has taken place in the Department of Biostatistics (DB) of a Research Center. The QIP was carried out by the team comprised of DB quality officer, quality management specialist and head of the quality department. The team was led by DB’s quality officer. Ethical approval was granted by the Institutional Review Board. A PI’s team is in general comprised of co-investigators, research coordinators/research assistants. The PI submits the clinical research project for study proposal processing, ethical approval, SDA to the research center. The DB offers SDA services to PIs of multidiscipline through a consulting process. The PI submits collected data for SDA to DB, and the department conducts statistical analysis and generates results. The DB keeps historical records in its knowledge base system for all submitted datasets for SDA. The QIP was conducted in two stages.

### Baseline measurement

In pre-intervention stage, 45 datasets were selected from DB knowledge base system from the preceding two years, using following inclusion criteria: (i) Datasets that were designed and collected primarily for a given clinical research project and generated by the PI’s team (ii) The included study types were longitudinal studies with multiple data capturing points (i.e. cohort/case–control/randomized controlled trials), or a single point of data capturing as in cross-sectional studies (iii) Only datasets where data was entered manually by humans. Data generated using electronic data collection forms and student projects were excluded. The primary outcome was defined as the number of defects per dataset.

### Defining data quality metrics

Several methods have been described for defining DQ. DQ is the conformance to best practices for data management. Achieving 100% DQ is possible but not practical.

A 12-item checklist to assess the datasets accuracy was developed based on the six DQ domains (i) completeness (ii) uniqueness (iii) timeliness (iv) accuracy (v) validity and (vi) consistency [18]. The twelve items were the top data errors that have been identified from review of historical datasets prior to SDA. The datasets were considered *defective* if they had one or more of the following defects: missing codebook (data dictionary); if codebook was not submitted with the dataset for SDA; inconsistencies within the codebook and the dataset; inaccurate format; unanalyzable data structure; missing outcome variables; missing analytic variables; missing values; uncoded values; mis-coded values; embedded values; implausible values; and unformatted values [19]. The degree of dataset conformance was defined as *‘unacceptable’* if the identified defects were: missing codebook at the time of data submission for SDA; inconsistencies within the codebook and dataset; inaccurate format; unanalyzable data structure; or missing outcome variables. The unacceptable datasets were not accepted for SDA till the missing documentation was completed and inaccurate format was adjusted. The dataset was considered *‘sub-optimal’* if it had any of the following data entry errors: un-coded values; miscoded values; missing values; implausible values; embedded values; unformatted variables; and missing analytic variables. The sub-optimal datasets were accepted for SDA; however required data cleaning prior to SDA. A score of one was assigned to each single defect in the checklist, which resulted in total score between zero and twelve.

### Metrics evaluation framework

The DMAIC (Define, Measure, Analyze, Improve, Control) framework was applied to conduct the project. The six sigma improvement tools were used: SIPOC; KANO Model; Defects per Unit (DPU), Defects per Million Opportunities (DPMO); Yield; Sigma; Root and Cause Analysis; PARETO; Cause and Effect Matrix; and Failure Modes and Effect Analysis (FMEA) [20–23].

The table given below summarizes the metrics and calculations:

**Table:** 

Metrics	Formula	Description
1. SIPOC (supplier, input, process, output, customer):	–	Identifies all elements of a process improvement before measuring baseline
2. DPU	$\frac{\mathrm{Number}\ \mathrm{of}\ \mathrm{defects}\ \mathrm{observed}}{\mathrm{Number}\ \mathrm{of}\ \mathrm{units}\ \mathrm{inspected}}$	Provides a measurement of the average number of defects in a single unit [23, 24].
3. DPO	$\frac{\mathrm{Number}\ \mathrm{of}\ \mathrm{defects}\ \mathrm{observed}\ \mathrm{in}\ \mathrm{a}\ \mathrm{unit}}{\mathrm{Number}\ \mathrm{of}\ \mathrm{opportunities}\ \mathrm{of}\ \mathrm{error}\ \mathrm{in}\ \mathrm{a}\ \mathrm{unit}}$	Measures the number of defects that occur per opportunity for success or failure [23, 24].
4. DPMO	DPO ∗ 1000, 000	“Total number of defects observed divided by the total number of opportunities expressed in events per million, sometimes called defects per million” [23, 24].
5. Yield	$Yield\kern0.5em \%=\frac{\left(1- DPO\right)}{100}$	“Traditionally, yield is a proportion of correct items (conforming to specifications) you get out of the process compared to the number of raw items put into it” [21, 23, 24].
6. Sigma		Six sigma quality performance means 3.4 defects per million opportunities [21]. The term sigma in six sigma refers to the standard deviation, which is a measure of variability in a process [20].

Pareto chart was used to identify the major types of defects in the datasets. Cause and Effect Matrix was used to identify the factors affecting the QIP outcome. FMEA was used to identify the possible ways the key process of data generation can go wrong and to identify possible actions to minimize/eliminate failures. The identified potential failures based on FMEA were; selection of key variables, development of incomplete data collection sheet, missing/inaccurate codebook, inaccurate data structure, missing data, and data results reporting. The data collected were entered into an Excel® spreadsheet (Microsoft Corp., Redmond, WA, USA) for SDA.

### Mode of intervention

Based on a focus group discussion with the QIP team, and data analysts, possible solutions to improve the DQ were proposed. To select the most effective interventions, a Decision Matrix (DM) was built based on the selection criteria: (i) feasibility of the solution; (ii) ease of implementation; (iii) cost of proposed solution; (iv) staff involved; and (v) time taken to implement the solutions. Different weightage has been assigned based on rating and scores, for each item in the DM considering the prior experience and organizational policies.

The final selected mode of interventions included the following:

- A data completion checklist (which comprised of the twelve items based on the commonly encountered data errors from the historical data review)
- An introductory package for PIs (which included a data structuring guide, data structuring Excel® templates, a codebook sample, and brochures for the required data elements and variables based on study type).
- Training of research coordinators involved in a research project.
- Video materials focusing on how to structure and enter data in excel sheets.
- Electronic data capturing using electronic case report forms.

The introductory package was disseminated at any point of contact with the PIs; either at the time of study proposal submission, or while consulting with the DB. The PI who has consulted more than one time with the DB and had an experience of SDA was considered as a *‘returning principal investigator’*.

### Statistical analysis

Pre–Post analysis was performed to compare data defects after the implementation of the selected mode of interventions. The association between defective data with presence of a research coordinator on board and returning PIs was analyzed by using Fisher’s exact test. The association between defective data and the nature of data capturing (longitudinal vs. cross-sectional) was analyzed by using Fisher’s exact test. Pre-Post change in defects (zero, one, two or more defects) was compared by using chi-square test. Significance was declared at an α value of < 0.05. Analyses were performed using SPSS (IBM SPSS Statistics for Windows, Version 24.0 IBM Corp.)

## Results

### Baseline results

**Fig. 1:**
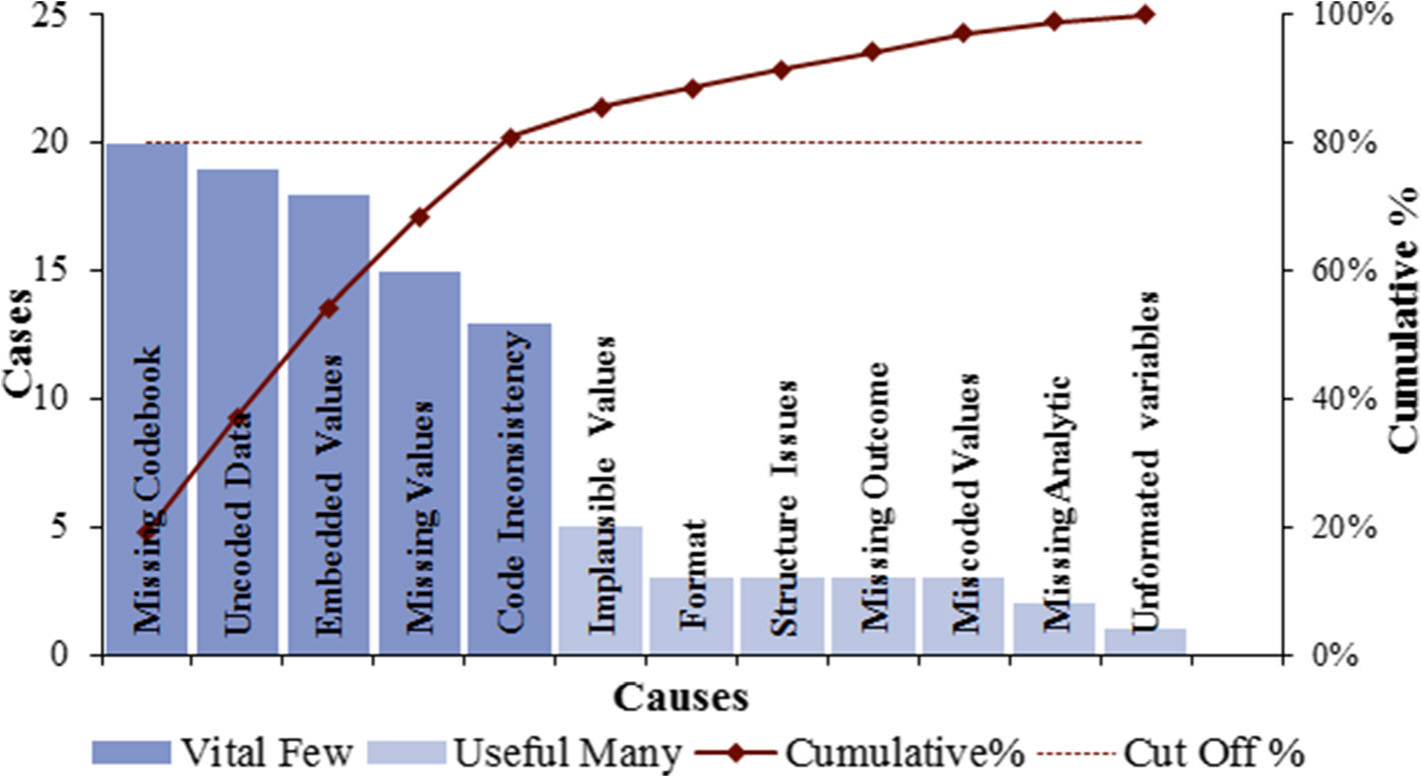
PARETO Chart of Most Common Types of Data Defects at the Baseline

Forty-five datasets submitted for SDA were included at baseline. Figure 1 displays the Pareto chart highlighting the cumulative percentages of the most common types of defects. The PARETO result shows that missing codebook and data entry errors (un-coded data, embedded values, missing values, and coding inconsistencies) in total represent 80%. However missing codebook alone represents (20/45) 44.4%. Twenty-four (53.3%) datasets were longitudinal studies that captured data at multiple time points, whereas 21(46.6%) datasets were cross-sectional studies that captured data only at one time point. Ten (22.2%) datasets were submitted by the PIs who had prior SDA. Seventeen (37%) datasets came through PIs’ team involving a research coordinator. At baseline six (13.3%) datasets had zero defects, eight (17.8%) had one defect, and 31 (69%) had two or more defects (Table 1). The DPMO value was 194,444.44, the Yield value was 80.55, and the Sigma value was 2.4. The associations between defective data and the presence of a research coordinator (*p* = 0.251), and returning PI (*p* = 0.113) were not significant, whereas the nature of data capturing; single data point vs multiple data points was significantly associated with data defects (*p* = 0.007; Table 1).

**Table 1:** Pre vs Post-Intervention Change in DPU, DPMO, Yield and SIGMA

Measures	Pre-Intervention *n = 45*		Post-Intervention *n = 21*	
Distribution of defects				
Zero defects	6 (13.3)		17 (81)	
One defect	8 (17.7)		2 (9.5)	
Two or more defects	31 (69)		2 (9.5)	
DPU (defects per unit)	2.33		0.57	
DPMO (defects per million opportunities)	194,444.44		47,619.04	
Yield	80.55		95.23	
SIGMA	2.4		3.2	
	Pre-intervention datasets		Post-intervention datasets	
	Not Defective	Defective	Not Defective	Defective
Data Capturing Points *n* (%)				
Single data capturing point (Cross-Sectional studies)	6 (28.6)	15 (71.4)	8 (66.7)	4 (33.3)
Multiple data capturing points (Longitudinal studies)	0	24 (100)*	9 (100)	0
Research Coordinator on board *n* (%)				
No	5 (17.9)	23 (82.1)	7 (63.60)	4 (36.4)
Yes	1 (5.9)	16 (94.1)	10 (100)	0
Principal Investigators (PIs) Requesting Data Analysis *n(%)*				
Returning PIs	3 (30)	7 (70)	14 (100)	0
New PIs	3 (8.6)	32 (91.4)	3 (42.9)	4 (57.1)**

Longitudinal studies = Cohort/Case-Control/Randomized controlled trials (RCT)

The reported percentage is row percentage

*p* -value is based on Fisher's exact test

* *p* = 0.007

**p = 0.006

**Fig. 2:**
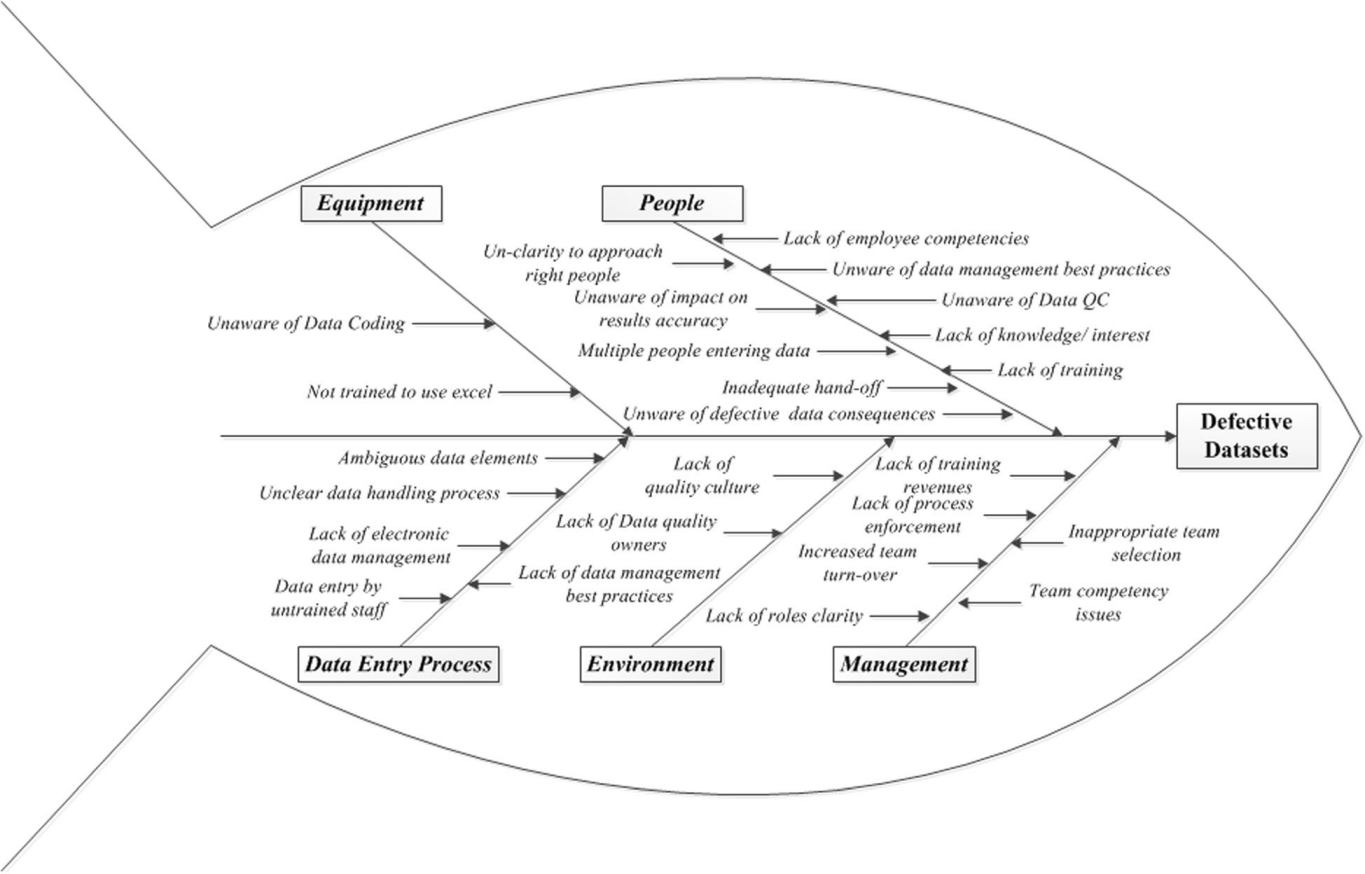
Root Cause Analysis of Data Defects

The Root and Cause analysis is summarized in Fig. 2. The reasons identified for defective datasets were lack of training, inadequate hand off, lack of process enforcement, lack of role clarity, lack of data owners, lack of employee competencies, and poor awareness of best practices for data coding and management.

### Post-intervention results

Twenty-one datasets were submitted for SDA after implementation of the selected modes of interventions. Ten (47.6%) datasets came through PI’s team involving a research coordinator. Twelve (57.1%) datasets were longitudinal studies that captured data at multiple time points, whereas nine (42.8%) were cross-sectional studies that captured data at only one time point. Fourteen (66.66%) datasets were submitted by the PIs who had prior SDA. The associations between defective data and the presence of a research coordinator (*p* = 0.08) and nature of data capturing; single data point vs multiple data points (*p* = 0.104) were not statistically significant.

**Fig. 3:**
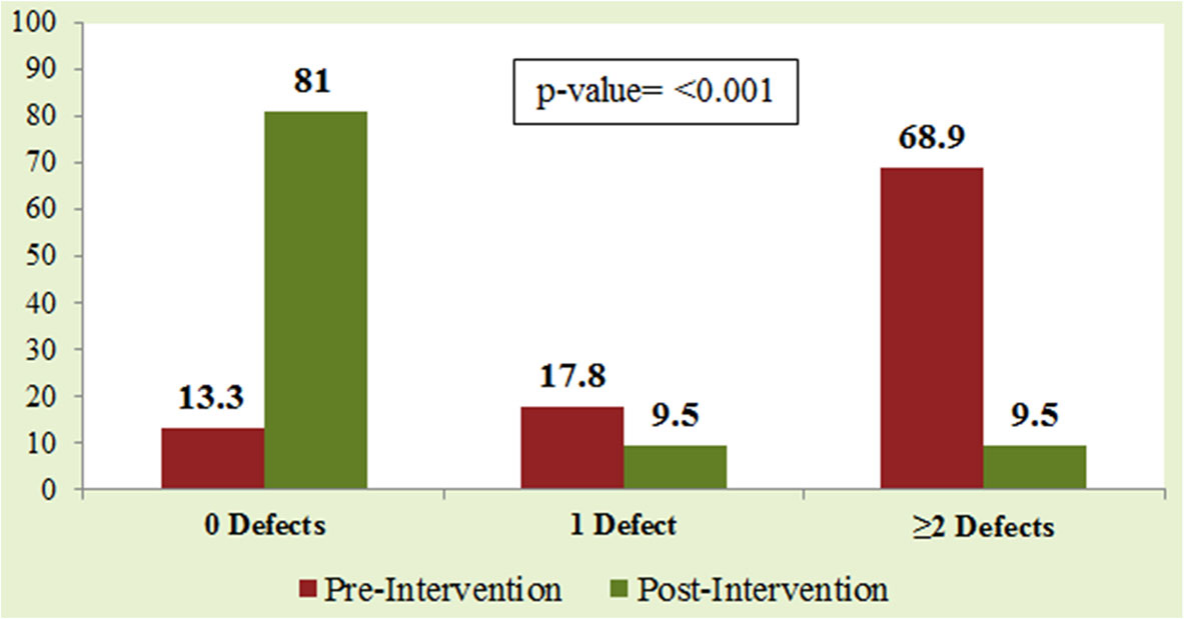
Pre vs Post Intervention Change in Number of Data Defects

**Fig. 4:**
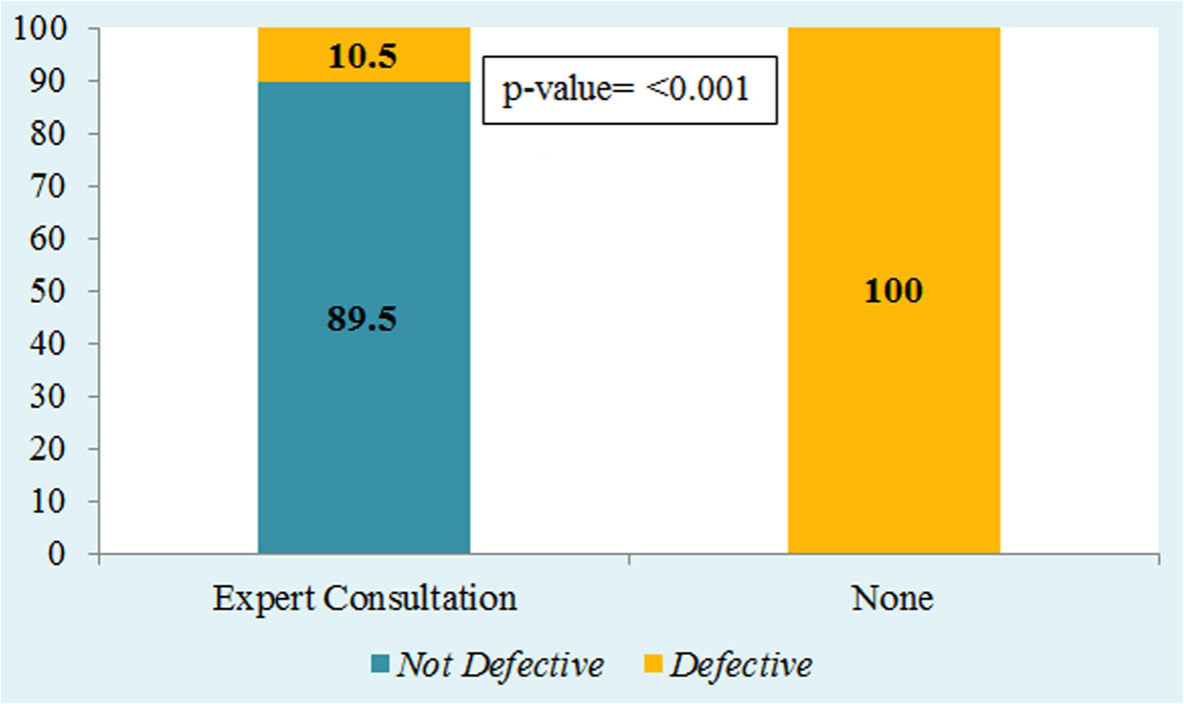
Association of Expert Consultation with Defective Datasets

Defective data was significantly associated with the PI consulting first time with the DB or had a prior SDA (*p* = 0.006; Table 1). Seventeen (81%) datasets had zero defects, two (9.5%) had one defect, and two (9.5%) had two or more defects. The DPMO value was 47,619.04, the Yield value was 95.23, and the Sigma value was 3.2 (Table 1). The change in data defects was observed after the implementation of modes of intervention. The proportion of datasets with zero defects had increased from 13.3 to 81%, whereas the proportion of datasets with two or more defects had decreased from 69 to 9.5%, (*p* = < 0.001; Fig. 3). Post-intervention, 11(52.3%) datasets were generated by using electronic data capturing. 19(90%) datasets had undergone consultation with data experts prior to structuring their datasets. Of them, only two (10.52%) datasets were defective compared with datasets that had received no consultation prior to data structuring (*p* = < 0.001; Fig. 4).

## Discussion

The purpose of this paper was to address the importance of DQ and conformance with best practices for data management in clinical research studies. Both data collection and entry can impact the expected outcome of a dataset. Thus, minimizing data entry errors is of paramount importance. The main focus of this paper was data entry errors, because the conclusion established from a dataset largely depends upon data entry [25]. DQ is a fundamental aspect of any clinical research study. To the best of our knowledge, this is the first QIP to focus on DQ in clinical research studies using pre–post analysis after the implementation of modes of intervention. The results of current QIP show a change in the distribution of data defects after the interventions. The proportion of datasets with zero defects had increased from 13.3 to 81%, whereas that of datasets with two or more defects had decreased from 69 to 9.5%, which shows significant improvement post-implementation of the suggested solutions.

The first survey to identify obstacles to good DQ was conducted by Haug et al. (2011). The foremost barrier to good DQ identified was “lack of delegation of responsibilities” [26]. Haug et al. (2011) stated that “data quality research has not yet advanced to the point of having standard measurement methods for any of these issues” [27]. In the current QIP, the main causes of defective datasets identified by Root and Cause analysis were; lack of training, inadequate hand off, lack of process enforcement, lack of role clarity, lack of data owners, lack of employee expertise, and poor awareness of best practices for data coding and management. The potential barriers to good DQ in clinical research datasets identified in the current QIP are similar to those reported over the years by researchers; “lack of delegation of responsibilities, lack of employee competencies, and lack of master data management” [26]. These reported barriers to DQ are also similar to those reported in the telecommunications industry; “lack of roles and responsibilities, lack of data quality owners, inefficient organizational procedures, lack of reward; and disregard of administrative details, e.g. staff training, job descriptions, and communication or administrative issues” [28].

The selection of intervention modes were based on the common DQ barriers as identified on the root cause analysis. The DQ can be improved by using electronic data collection, as reported in a recent study where electronic data collection forms have resulted in lower error rates (missing values/unreadable values) compared to paper based forms [29]. In post-intervention stage 52% datasets were generated by using electronic data capturing. The electronic data capture yields data of higher quality and is one of the suggested solutions to improve poor DQ [29]. The study team re-training has been reported as a solution as well [30]. The results indicate that longitudinal studies involving data capturing at multiple time points (cohort/case–control/randomized controlled trials) tend to have more data defects. In the current QIP an objective assessment of DQ was conducted. DQ was assessed based on DQ metrics [12], and DQ metrics more specifically was based on the type of study and nature of data capturing. The findings of increased defects in datasets involving data capturing at multiple given points is supported by the results of a study by Whitney et al. (1998). Data capturing in longitudinal studies further impacts the quality of the datasets generated through factors such as multiple data points over time, changes in measurements over time, and staff turnover. The quality can be further jeopardized in datasets generated by multicenter studies [25].

It was speculated that the presence of a research coordinator would reduce the defects in a dataset. However, the inclusion of a research coordinator in a clinical research project had no significant effect on DQ. One possible reason for this is that data are generally handled by multiple team members and not exclusively by a research coordinator. In addition, the number of defective dataset did not differ between datasets with returning or a new PI. This is probably due to the variability of the study team of each project. The results also indicated that consultation with data experts prior to structuring datasets improved DQ.

## Conclusion

Research study teams often have limited knowledge of data structuring, entry, and coding or the impact of DQ on results. Given the need for good DQ in clinical research studies, we recommend training programs, compliance with data management best practices, early consultation with data experts and electronic data capturing to improve DQ. Moreover, development of data quality metrics is necessary for multicenter and longitudinal projects [31, 32].

## Abbreviations

DB: Department of Biostatistics
DM: Decision matrix
DMAIC: Define, Measure, Analyze, Improve, Control
DPMO: Defects per million opportunities
DPU: Defects per unit
DQ: Data Quality
FMEA: Failure mode and effect analysis
PIs: Principal investigators
QIP: Quality improvement project
SDA: Statistical data analysis
